# Prognostic significance of EGFR and Her-2 in oral cavity cancer in betel quid prevalent area cancer prognosis

**DOI:** 10.1038/sj.bjc.6601171

**Published:** 2003-08-12

**Authors:** I-H Chen, J T Chang, C-T Liao, H-M Wang, L-L Hsieh, A-J Cheng

**Affiliations:** 1 Department of Otorhinolaryngology, Section of Head and Neck Surgery, Chang Gung Memorial Hospital,Taoyuan, Taiwan; 2 Department of Radiation Oncology, Chang Gung Memorial Hospital, Taoyuan, Taiwan; 3 Division of Hematology/Oncology, Department of Internal Oncology, Chang Gung Memorial Hospital, Taoyuan, Taiwan; 4 Department of Public Health, Chang Gung University, Taoyuan, Taiwan; 5 School of Medical Technology, Chang Gung University, Taoyuan, Taiwan; 6 Taipei Chang Gung Head and Neck Oncology Group, Taoyuan 333, Taiwan

**Keywords:** oral cancer, EGFR, Her-2, prognosis, betel quid

## Abstract

Although several studies have found overexpression of epidermal growth factor receptor (EGFR) proteins EGFR and Her-2 in head and neck cancers, the clinical relevance of the finding varies. We examined the expression and clinical association of these molecules with oral squamous cell carcinoma in an area where betel chewing is prevalent. EGFR and Her-2 proteins were measured in 59 paired (grossly normal and cancer) tissues by an enzyme immunoassy method. The cutoff value for gene overexpression was defined as the level of mean expression in normal tissue plus two s.d. A total of 59% of the patients consumed alcohol, 90% smoked tobacco, and 90% chewed betel quid. Of the patients assayed, 34 (58%) and 24 (41%) had EGFR and Her-2 overexpression, with average 3.5- and 1.5-fold elevations. EGFR overexpression has been shown to be statistically associated with T stage, N stage, overall TMN stage, primary tumour depth, lymph node extra-capsular spread, and poor survival. Her-2 overexpression, however, did not demonstrate a similar association with clinicopathological parameters or therapeutic outcome. On multivariant analysis, EGFR overexpression (*P*=0.041) and N stage (*P*=0.024) were the only independent factors for overall survival. These results indicate that the molecular targeting therapy to EGFR may be a treatment for oral cavity cancer in the betel quid-chewing prevalent area.

Squamous cell carcinoma (SCC) of the oral cavity is one of the most frequent cancers in the world (Vokes *et al*, 1993). This disease occurs much more frequently in males (Johnson, 1991). Epidemiologic studies show a strong association between its incidence and environmental carcinogens, especially the use of tobacco, alcohol, and betel quid (Franceschi *et al*, 1990; Ko *et al*, 1995; Hsieh *et al*, 2001). In Taiwan, the incidence of oral carcinoma is one of the highest in the world. The incidence of oral cavity cancer was 20 per million in our male population, comprising approximately 4–5% of all malignancies (Chen, 1987). Approximately 85% of all oral cavity cancer patients habitually use betel quid (Chong, 1966). The majority of oral carcinoma in Taiwan occurs in buccal mucosa (ICD145), which was relatively less common in Western populations (Daftary *et al*, 1991). Such geographical differences in incidence and cancer sites may result from exposure to different carcinogens, and possibly also from genetic predisposition.

The overall 5-year survival rate for patients with oral carcinoma is among the lowest of the major cancers and has not changed during the past two decades (Parker *et al*, 1996). The standard treatment for patients with this cancer is surgery, radiation, or multiple modalities for patients at high risk (Clayman *et al*, 1996). Although standard care is frequently initially successful in early-stage cancer (stage I or II), disease relapses still occur in about 20–30%, particularly local tumour or lymph node recurrence (Vikram, 1994; Clayman *et al*, 1996). For patients with advanced oral carcinoma (stage III or IV), standard therapy is far less successful. The recurrent rate in advanced stage is approximately 50–60% and distant metastasis is 20–35% (Clayman *et al*, 1996). Even if there is a good treatment response, patients with advanced disease often suffer substantial functional and cosmetic morbidity, which decreases the quality of life. The identification of prognostic factors that may affect disease outcome may lead to improvements in adjuvant systemic therapy and better control of the disease.

The tyrosine kinase receptor, epidermal growth receptor (EGFR) family proteins EGFR and Her-2 have been reported to be overexpressed in many cancers. They are often associated with a poor prognosis, suggesting that they are potential molecular targets for anticancer therapy. This family of proteins consists of four closely related transmembrane receptors, including EGFR (erbB1), HER-2 (erbB2), HER-3 (erbB3), and HER-4 (erbB-4) (Olayioye *et al*, 2000; Simon, 2000). Several ligands, such as EGF and amphiregulin, bind to EGFR, whereas there is no known high-affinity ligand binding to Her-2. However, both EGFR and Her-2 interact with other members of the family by heterodimerisation, resulting in activation of their intrinsic kinase activity (Olayioye *et al*, 2000). Overexpression of EGFR and Her-2 have been reported to be associated with higher grades or reduced survival in a variety of cancers, including breast, colorectal, and head and neck cancers (for a review, see Klijn *et al*, 1992; Salomon *et al*, 1995). Several molecular therapeutic agents against EGFR or Her-2, such as Cetuximab and Herceptin, have been studied in clinical trials (Colomer *et al*, 2001; Robert *et al*, 2002).

Although several studies have found overexpression of EGFR and Her-2 in head and neck cancers, the clinical relevance of the finding varies. For example, Storkel *et al* (1993) found that overexpression of EGFR was associated with shortened survival, but Werkmeister *et al* (2000) reported that Her-2 was strongly associated with survival. Christensen *et al* (1995) and Khan *et al*, (2002) could not find significant correlation of either EGFR or Her-2 with clinicopathological features or prognosis; however, Bei *et al* (2001) and Xia *et al* (1999) found colocalisation of both molecules in oral cancer tissues and the combined use of these molecules is a stronger predictor for the cancer prognosis. We therefore designed this study to investigate EGFR and Her-2 in paired grossly normal and cancerous tissues from squamous cell carcinoma of oral cavity patients. Our aims were to determine their levels of expression and see if these levels correlated with clinicopathological variables, and if they were useful prognostically.

## MATERIALS AND METHODS

### Patients, tissues and cells

Fifty-nine consecutive patients seen in 1999 at the Otorhinolaryngology or Head and Neck Surgery clinics at Chang Gung Memorial Hospital (Taoyuan, Taiwan) were enrolled for the study. Written informed consent was obtained from all patients participating in this study. A questionnaire was filled out by each patient before the first clinical visit investigating whether or not the patient was a habitual betel net chewer (daily chewer), cigarette smoker (daily smoker), and/or regular alcohol drinker (daily drinker). The standard treatment was radical surgery for early-stage patients and adjuvant radiotherapy for patients with intermediate risk, such as a close margin or lymph node metastases. Concomitant chemoradiotherapy was given in patients with lymph node metastases and extracapsular spread (ECS). All cancers were histologically graded as well differentiated, moderately differentiated, or poorly differentiated, according to the World Health Organization (WHO) classification (Shanmugaratnam, 1991). For each sample, the presence of bone or nerve invasion, lymphatics, blood vessels, tumour depth, and the presence or absence of lymph node ECS were specifically recorded. Tumour pathological staging was classified according to the AJCC system (Fleming *et al*, 1998). Biopsies of cancerous and grossly normal mucosa tissue were obtained from each subject before chemo- or radiotherapy. A portion of each tissue sample was stored in liquid nitrogen until use for molecular assay. An oral cancer cell line OC2 (Wong *et al*, 1990) was used as a positive control. OC2 cells were grown at 37^o^C, 5% CO_2_ in RPMI medium containing 10% fetal bovine serum and antibiotics (100 U ml^−1^ penicillin, 100 Uml^−1^ streptomycin, and 0.25 *μ*g ml^−1^ amphotericin B).

### Extraction of cellular proteins

The investigators were blinded as to the source and type of tissue being assayed. Tissue samples (∼50 mg) were homogenised in 300 *μ*l of a lysis buffer (10 mM Tris-HCI, pH 7.5, 1 mM MgCl_2_, 1 mM EGTA, 0.5% CHAPS (Pharmacia Ontario, Canada), 10% glycerol, 5 mM mercaptoethanol, and 0.1 mM phenylmethylsulphonyl fluoride (PMSF) in Kontes tubes with matching pestles rotated at 450 r.p.m. After 30 min at 4°C, the lysate was centrifuged at 15 000 r.p.m. for 30 min at 4°C. The supernatants of the protein extracts were used for the EGFR and Her-2 ELISA assay. The protein concentration of each tissue sample was determined using Coomassie protein assay reagent (Pierce).

### Determination of EGFR and Her-2 protein levels

An enzyme-linked immunosorbent assay (ELISA) was used to detect tissue EGFR and Her-2 protein expression. The ELISA kits were purchased from CalBiochem Inc. (CA, USA). A total of 10 *μ*g cellular protein was used in each assay, performed according to the manufacturer's protocol. All samples were analysed in duplicate and the average of the two was recorded. To define the relative expression of EGFR or Her-2, both cancer tissue and the normal counterpart samples were assayed. The cutoff value for gene overexpression was defined as the level of mean plus two s.d. in the normal tissue expression values, and was designated as one-fold of overexpression.

### Statistical analysis

The Pearson *χ*^2^ test was used to look for the association between EGFR or Her-2 expression and clinicopathological parameters, including tumour extent (T, N, and overall stage) and the pathological findings (degree of differentiation, tumour depth, or ECS). For prognostic factors analysis, the Kaplan–Meier method was used for single-variant analysis and the Cox logistic regression model was used for multivariant analysis. All *P*-values presented were two-sided, and the significance level was set at <0.05.

## RESULTS

### Patient characteristics

The patient characteristics are summarised in Table 1

**Table 1:** Characteristics of patients with oral cancer

**Characteristic**	**Number**	**Percentage (%)**
Total	59	100
Sex		
Female	0	0
Male	59	100
Age		
⩽40 years	13	22
41–60 years	31	53
>60 years	15	25
Habits		
Alcohol drinking	35	59
Smoking	53	90
Betel quid chewing	53	90
Cancer site		
Buccal mucosa	24	41
Tongue	18	31
Others	17	29
Cancer histological grade		
Well differentiated	20	34
Moderately differentiated	33	56
Poorly differentiated	6	10

. Their median age was 48.0 years, ranging from 31 to 78, and all were male. A total of 59% of the patients consumed alcohol, 90% smoked tobacco, and 90% chewed betel quid. Cancers included 24 (41%) in the buccal mucosa, 18 (31%) in the tongue, and 17 (29%) in other sites. All cancers were SCC, with 20 (34%) well differentiated, 33 (56%) moderately differentiated, and six (10%) poorly differentiated. The disease staging is summarised in Table 2

**Table 2:** Tumour and node stage distribution

**Stage**	**T1**	**T2**	**T3**	**T4**	**Total**
N0	7	15	4	11	37
N1	0	2	0	3	5
N2	0	7	5	5	17
					
Total	7	24	9	19	59

. Of the 59 patients, seven (12%) had stage I disease (T1N0), 15 (25%) had stage II (T2N0), six (10%) had stage III (T3N0, T2N1), and had 31 (53%) stage IV (T4N0, T4N1, T2N2, T3N2, T4N2).

### The distribution of EGFR and Her-2 in oral cancer tissues

**Figure 1:**
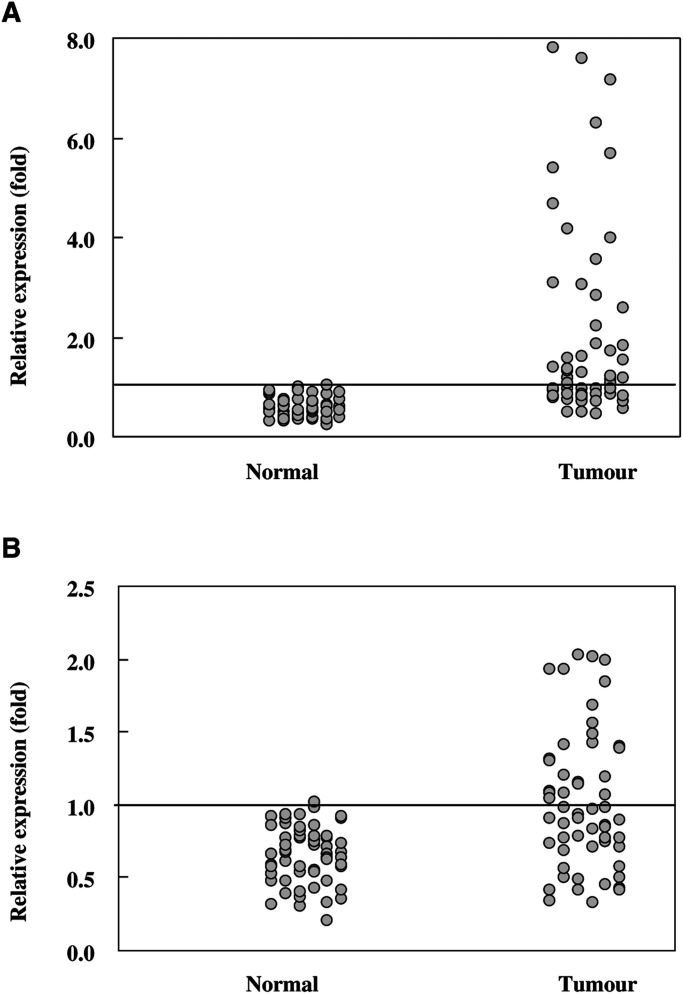
Relative expression of Her-2 and EGFR. (**A**) Relative expression of EGFR (**A**) and Her-2 (**B**) in normal and cancer tissue samples. The horizontal bar in the figure indicates the cut-off value (designated as one-fold)

The relative expression of EGFR and Her-2 in all 59 patients were examined and plotted in Figure 1

. Similar results were obtained when we assayed EGFR and Her-2 levels using different sites of the same tumour. As shown in the Figures, EGFR was overexpressed in two (3%) normal mucosa tissues and 34 (58%) cancer tissues. Similarly, Her-2 was overexpressed in one (2%) normal tissue and 24 (41%) cancer tissues. Compared to all normal samples, the average expression of EGFR in cancer tissue was 3.48-fold, with an s.d. of 2.01, while the average expression of Her-2 was 1.51-fold, with an s.d. of 0.45. Thus, although the levels of both EGFR and Her-2 expression differed significantly between normal and cancer tissues (*P*<0.001), EGFR had greater overexpression than Her-2 on average in all the cancer patients. The distribution of EGFR and Her-2 expression in the 59 oral cancer tissues is summarised in Table 3

**Table 3:** EGFR and Her-2 overexpression in oral cancer tissues

	**EGFR overexpression**		
**Her-2 overexpression**	**No**	**Yes**	**Total**
No	17	18	35
Yes	8	16	24
			
Total	25	34	59

. Of the patients, 17 (29%) had normally expressed in both molecules, 16 (27%) had overexpressed in both molecules, and 26 (44%) had overexpressed in either molecule. EGFR and Her-2 were not significantly coexpressed (*P*=0.245).

### Correlation of EGFR or Her-2 with clinicopathological parameters

The correlations of the expression levels of EGFR or Her-2 with clinicopathological parameters are shown in Table 4

**Table 4:** Association of EGFR and Her-2 status with clinicopathological parameters

	**EGFR overexpression**				**Her-2 overexpression**		
**Parameter**	***N***	**No(%)**	**Yes(%)**	***P*-value**	**No(%)**	**Yes(%)**	***P*-value**
T stage							
T1–T2	31	18 (58)	13(42)	0.010	17(55)	14 (45)	0.461
T3–T4	28	7 (25)	21(75)		18(64)	10(36)	
Nstage							
*N*=0	37	20 (54)	17 (46)	0.019	22 (60)	15 (41)	0.978
*N*>0	22	5 (23)	17(77)		13(59)	9 (41)	
Overall stage							
I–II	22	15 (68)	7 (32)	0.002	12 (55)	10 (46)	0.565
III–IV	37	10(27)	27(73)		23(62)	14(38)	
Differentiation							
Well	20	9 (45)	11 (55)	0.852	13 (65)	7 (35)	0.050
Moderate	33	13(39)	20(60)		16(49)	17(52)	
Poor	6	3(50)	3(50)		6(100)	0 (0)	
Tumour depth>10 mm							
No	26	15 (58)	11 (42)	0.035	13 (50)	13 (50)	0.196
Yes	33	10(30)	23(70)		22(67)	11(33)	
ECS of lymph node							
No	43	22 (51)	21 (49)	0.025	25 (58)	18 (42)	0.762
Yes	16	3(19)	13(81)		10(66)	6(38)	
							
Total	59	25(42)	34(58)		35(59)	24(41)	

. As shown in the table, significant correlations were found between EGFR expression and tumour extent (T stage) (*P*=0.010), lymph node status (N stage) (*P*=0.019), clinical overall stage (*P*=0.002), tumour depth (*P*=0.035), and ECS of lymph node (*P*=0.025). However, there was no association between Her-2 expression with cancer stage or any other clinicopathological parameters. The results indicate that EGFR has an association with the aggressiveness of oral cancer.

### Evaluation of possible prognostic factors associated with oral cancer

As shown in Table 5

**Table 5:** Univariant analysis of prognostic factors in oral cancer

**Parameter**	**Group**	**2-year survival (week)**	***P*-value**
T stage	T1-2/T3-4	73/22	0.101
N stage	N0-1/N2-3	80/22	<0.001
Overall stage	I–II/III–IV	86/51	0.008
Tumour depth(mm)	⩽10/>10	84/48	0.002
LN ECS	No/yes	79/19	<0.001
EGFR overexpression	(−)/(+)	88/45	0.001
Her-2 overexpression	(−)/(+)	64/62	0.928

, for the 2-year survival, there were significant correlations with N stage (*P*=0.000), overall stage (*P*=0.008), tumour depth (*P*=0.002), ECS of lymph node (*P*=0.000), and the expression levels of EGFR (*P* =0.001). To define the role of the above factors further, multivariant analysis was conducted and has been demonstrated in Table 6

**Table 6:** Multivariant analysis of prognostic factors in oral cancer

**Parameter**	**Risk ratio**	**95% CI^a^**	***P*-value**
T stage	0.781	0.22–2.36	0.583
N stage	4.215	1.21–14.74	0.024
Overall stage	0.601	0.009–4.16	0.606
Tumour depth	3.854	0.87–17.16	0.077
ECS of lymph node	1.083	0.52–3.07	0.601
EGFR overexpression	5.882	1.07–32.31	0.041
Her-2 overexpression	1.083	0.36–3.26	0.888

a CI=confidence interval.

**Figure 2:**
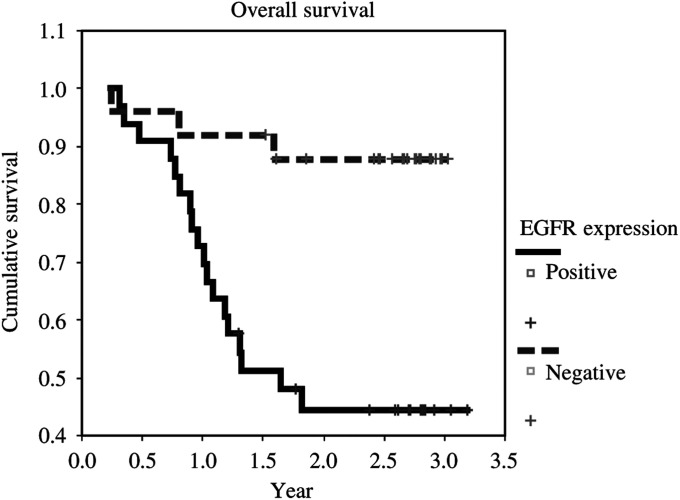
Kaplan–Meier overall survival curves according to EGFR overexpression.

. Lymph node metastasis (*P*=0.024) and overexpression of EGFR (*P*=0.041) were the only independent variables associated with poor survival, with a risk ratio of 4.22 for lymph node metastasis (95% CI=1.21–14.74), and 5.88 for EGFR overexpression (95% CI=1.07–32.31). The Kaplan–Meier overall survival curves related to EGFR overexpression are shown in the Figure 2

.

## DISCUSSION

In our study, we found EGFR and Her-2 to be differentially expressed in oral SCC. Consistent with other reports, we found that both EGFR and Her-2 were overexpressed in a subset of oral cancer patients (58 and 41%, respectively). However, compared to Her-2, EGFR overexpression was more significant in terms of the expression level (3.5-fold *vs* 1.5-fold). Although these two molecules were coexpressed in some patients, this was not a statistically significant association (*P*=0.245). A high level of EGFR, but not of Her-2, was strongly associated with tumour aggressiveness and poor survival.

Although some of our findings are consistent with other reports, we noted above the conflicting data produced by various authors. These differences may be due to differences in assay techniques. For example, most of the investigators used the immunohistochemistry method to examine protein expressions (Storkel *et al*, 1993; Xia *et al*, 1999; Bei *et al*, 2001; Khan *et al*, 2002), while we used the ELISA method to quantitatively analyse EGFR and Her-2 protein levels. The advantage of the immunohistochemistry method is the precise localisation of the protein molecules in cells. However, this method reported that data determined by microscopic examination may be influenced by the subjective assessment through different individuals. Although the ELISA technique is less used in clinical study, this method is also widely accepted to determine protein expression levels. Examples include using this technique in the studies of breast cancer, cervical cancer, lung cancer as well as head and neck cancer (Christensen *et al*, 1995; Pfeiffer *et al*, 1998; Contreras *et al*, 2002; Widschwendter *et al*, 2002). The key advantage of the ELISA method is to provide a quantitative result with relatively less bias. When comparing these two methods, Pfeiffer *et al* (1998) have reported that significant correlation was found in the quantification of EGFR or Her-2 levels between ELISA and immunohistochemical methods studied in the lung and in breast cancers. These studies suggest that comparable evaluation results may be obtained by using these two assay techniques.

Patient sampling is another variable between other studies and ours. Most investigators examine protein expressions between different tumour tissues (but no grossly normal counterpart tissues to compare) (Storkel *et al*, 1993; Xia *et al*, 1999; Bei *et al*, 2001; Khan *et al*, 2002). We analyse EGFR and Her-2 protein levels in the paired grossly normal mucosa and cancer tissues obtained from the same patients. These results will provide clearer information regarding the protein level changes after cellular transformation. Additionally, most other reports have evaluated populations in the West (Xia *et al*, 1999; Werkmeister *et al*, 2000; Bei *et al*, 2001), whereas ours focused on Southeast Asians. Since both carcinogen exposure (including betel quid chewing) and possible genetic predisposition vary between different geographic areas, the reported differences in EGFR or Her-2 expression may reflect different oral carcinogenic pathways in different populations.

In the upper aerodigestive tract, significant exposure of the mucosa surface to the same carcinogens or stimulants (such as alcohol or cigarettes) may lead to a multitude of somatic changes that are susceptible to the development of multiple primary cancers. This phenomenon is called ‘field cancerisation’ (Slaughter *et al*, 1953). Recent molecular studies have shown that genetical alterations could be found in different areas of histological normal mucosa. Examples are mutations of the p53 tumour suppressor gene and deletions on the short arm of chromosome 3 (Li *et al*, 1994; Van Dyke *et al*, 1994). In our grossly normal mucosa tissues, which provide an internal control for comparing them with cancer tissues in the same patient, they may not represent true normal samples, particularly in patients who drink, smoke, and chew betel quids. However, in our present study, there is no statistical difference of the protein expression levels of EGFR and Her-2 between the normal tissues from patients with or without exposure to tobacco, alcohol, betel quid, or the combined exposure (data not shown). These results suggest that the ‘filed’ effect of EGFR and Her-2 molecules on the surrounding oral normal mucosa is minimum. Apparently, together with multiple injuries and cellular genetic mutations on a specific tissue, a process described as ‘multistep carcinogenesis’ will eventually transform the cell into malignant cancer (Vogelstein et al, 1998).

For a molecule to be a good candidate as a target for anticancer therapy, several criteria must be fulfilled. First, the protein should be overexpressed in cancer tissues compared to normal tissues. Second, overexpression of the protein should be associated with a poor prognosis, which suggests that manipulation of the protein may result in alteration of the prognosis. In this study, we found that the membrane protein EGFR had both these characteristics. Our results do not support similar targeting of the Her-2 protein, even though it is commonly overexpressed in oral SCC. Recently, targeting of EGFR as a molecular adjuvant therapy has been clinically tried in head and neck cancer (Shin *et al*, 2001; Robert *et al*, 2002). This study provides a fundamental knowledge base, suggesting that targeting this molecule might be useful in betel quid-associated oral cancers (Shin *et al*, 2001).
